# The Pathogenetic Role of Melatonin in Migraine and Its Theoretic Implications for Pharmacotherapy: A Brief Overview of the Research

**DOI:** 10.3390/nu14163335

**Published:** 2022-08-15

**Authors:** Anna Zduńska, Joanna Cegielska, Izabela Domitrz

**Affiliations:** 1 Department of Neurology, Bielanski Hospital, 01-809 Warsaw, Poland; 2 Department of Neurology, Faculty of Medical Sciences, Medical University of Warsaw, 01-809 Warsaw, Poland

**Keywords:** migraine, melatonin, specific receptors for melatonin

## Abstract

Migraine is a chronic disease of global concern, regardless of socio-economic and cultural background. It most often and intensely affects young adults, especially women. Numerous mechanisms of a migraine attack have been identified (disturbances in the reaction of vessels, functions of neurotransmitters, cortical neurons, ion channels, receptors, the process of neurogenic inflammation), and many of its symptoms can be explained by activation of the hypothalamus and disturbances in its communication with other brain regions (including the brainstem). Numerous neuropeptides and neurochemical systems also play a role in migraine. One of them is melatonin, a hormone that allows the body to adapt to cyclically changing environmental and food conditions. In this article, we present the pathophysiological basis of melatonin release from the pineal gland and other tissues (including the intestines) under the influence of various stimuli (including light and food), and its role in stimulating the brain structures responsible for triggering a migraine attack. We analyze publications concerning research on the role of melatonin in various headaches, in various stages of migraine, and in various phases of the menstrual cycle in women with migraine, and its impact on the occurrence and severity of migraine attacks. Melatonin as an internally secreted substance, but also present naturally in many foods. It is possible to supplement melatonin in the form of pharmaceutical preparations, and it seems, to be a good complementary therapy (due to the lack of significant side effects and pharmacological interactions) in the treatment of migraine, especially: in women of childbearing age, in people taking multiple medications for other diseases, as well as those sensitive to pharmacotherapy.

## 1. Introduction

Migraine is a chronic and often lifelong disease that directly affects over a billion people of varying socioeconomic status in all regions and cultures of the world. Despite significant advances in diagnosis and treatment, migraine remains the second leading cause of disability worldwide. It reaches its greatest intensity in people aged 35–39, which makes it the main cause of disability worldwide in people under 50, especially in women [1].

The pathogenesis of migraine still remains unclear despite numerous theoretical concepts that have emerged over the past seventy years. The mechanism of a migraine attack includes pathological vasomotor regulation related to neuronal processes, changes in the neurotransmitter system, disturbances in the function of ion channels and many types of receptors, abnormal activity of cortical neurons as a trigger mechanism, and the process of neurogenic inflammation. All these factors play an important pathogenetic role, but their coherent combination still requires in-depth analysis [2,3].

The pathogenesis of migraine involves the neurobiological basis of cyclical cycles. Due to the occurrence of subsequent phases of a migraine attack i.e., prodromal symptoms, aura, headache with autonomic symptoms, postdromal and interictal period, which overlap or selectively occur in a particular patient, it is widely accepted that migraine is an inherited disorder of sensory processing, with many aspects of the underlying basis of this disorder. Neuroimaging studies have found hypothalamic activation and altered connectivity with other brain and brainstem regions that could explain many symptoms of migraine. Multiple neurotransmitters, neuropeptides, and neurochemical systems also play a role in migraine [4,5,6,7].

## 2. Migraine and Melatonin—Nutrition and Health Interactions

Melatonin is a hormone that allows the body to adapt to periodicity of changing environmental conditions, especially lighting and time of day. It is primarily produced by pinealocytes—the pineal glandular cells [8]. Suprachiasmatic nuclei in the hypothalamus, the function of which is governed by light signals transmitting through retinal ganglion cells, controls synthesis and secretion of melatonin [9]. Melatonin MT1 and MT2 receptors are G protein-coupled receptors expressed in various parts of the central nervous system (suprachiasmatic nuclei, cerebellar cortex, prefrontal cortex, hippocampus, substantia nigra, basal ganglia, ventral tegmental area, nucleus accumbens and retinal horizontal) and in peripheral organs (blood vessels, mammary gland, liver, bladder and kidney, ovary, prostate, testis, skin and the immune system) [10,11]. These receptors are also found in the gastrointestinal tract and are involved in regulating its motor activity, inflammation and pain. Melatonin found in the lower intestine comes mainly from intestinal sources, such as enterochromaffin cells throughout the guti, and to a small extent from parenteral sources such as the pineal gland. The intestine contains at least 400 times more melatonin than the pineal gland. After the detection of melatonin-synthesizing enzymes, *N*-acetyltransferase and hydroxyindole-O-methyltransferase in the intestines, the possibility of additional extra-ternal melatonin synthesis was considered. The melatonin produced in the gut is thought to act as a paracrine hormone that can be secreted both continuously and cyclically. Melatonin is also synthesized by many other extra-pineal cells, such as bone marrow cells, lymphocytes, mast cells, and epithelial cells, and it is unclear to what extent melatonin from these sources contributes to intestinal melatonin levels. The release of melatonin from all of these extra-thoracic sources appears to be independent of the photoperiod. In the gut, melatonin appears to play a significant role in regulating gut motility, the immune system, gastrointestinal secretion, and the release of energy-balancing peptides such as Y-peptide [12].

There is now evidence that melatonin may play a role in the biological regulation of circadian rhythms, sleep, mood and aging. Changes in melatonin levels have also been documented in cyclical seizure diseases such as migraine [13].

Although the mechanism of migraine remains unclear, most scientists agree that abnormal activation and sensitization of the trigeminovascular system play an important role in its pathophysiology [9]. Melatonin receptors have been found in the ganglia and nuclei of the trigeminal nerve, suggesting that melatonin weakens trigeminovascular nociception [14].

There are also other links between melatonin secretion and the pathophysiology of migraine. Research conducted over the last several decades has shown that melatonin is an effective antioxidant involved in the scavenging of free radicals and the regulation of enzymes involved in this process [9]. As a powerful endogenous radical scavenger, melatonin can directly scavenge excess free radicals. Melatonin is a more powerful antioxidant than glutathione, NADH, and vitamin C or E. In addition, melatonin is able to chelate toxic metals such as cadmium. It also stimulates the synthesis of other antioxidants, and induces the expression of gamma-glutamylcysteine synthetase (γ-GCS), the rate-limiting enzyme in GSH syn-thesis, in human vascular endothelial cells [15,16,17]. Melatonin through its immunomodulatory effect also plays a role in regulating the body’s immune response. Melatonin increases NK cell activity and the immune response through Th2 cells. It regulates the expression of the genes of several cytokines, including IL-2, IL-2R and IFN-γ released by human CD4 T cells [18]. In addition, melatonin causes a neuroimmunomodulatory effect on the immune system through its membrane receptors, which have been identified in the immune organs, tissues, bone marrow mononuclear cells (BMMNC) and leukocytes, and even subcellular compartments. Melatonin is effective in combating inflammation by various mechanisms, such as primary signaling pathways, modulation of related genes, and activation of membrane receptors. Melatonin exerts anti-inflammatory effects by inhibiting the signaling pathways of NF-κB-activated B cells. It also regulates the expression of some pro-inflammatory genes. In addition, it can inhibit the expression of inflammatory cytokines and chemokines [15,16,17].

By scavenging free radicals and inhibiting the release of inflammatory factors, melatonin can protect the brain from direct toxic effects and help maintain the structural and functional integrity of the brain by acting as a cell membranes stabilizer [9].

According to the neurogenic theory of migraine pathophysiology, the cause of migraine pain is neurogenic meningitis, resulting from the release of neuropeptides from the trigeminal nerve endings [19], especially substance P, nitric oxide and the calcitonin gene related peptide (CGRP) which are potent vasodilators and can activate nearby mast cells. Mast cells can then degranulate and release various vasoactive agents and chemoattractants such as histamine, intestinal vasoactive peptide (VIP) and nitric oxide, which in turn can recruit other immune cells, intensifying neurogenic inflammation and sustaining trigeminal nerve stimulation. Thus, mast cells play a key role in generating and maintaining dural inflammation. One of the most effective mast cell mediators is histamine, which has a vasodilating effect. It has been shown that an intravenous infusion of histamine can cause migraine headaches. Plasma histamine levels have also been shown to be elevated in people with migraine compared to those without headaches. Moreover, a diet without histamine helps to reduce the frequency of migraine attacks. These relationships underline the key role of histamine and mast cells in the pathophysiology of migraine. In the gastrointestinal tract, mast cells are usually present in the mucosa near peptidergic nerve fibers and endothelial cells and play a role in maintaining immunity. Mast cell degranulation can affect the enteric nervous system (ENS), gastrointestinal blood flow, and the blood-gut barrier (BGB). After mast cell activation, the released substances can cross the blood-gut barrier and enter the general circulation. Mast cells can be considered as a common pathophysiological factor linking migraine with the gastrointestinal tract [20]. 

Melatonin has an anti-inflammatory effect by inhibiting the synthesis of prostaglandin E2, involved with other substances in the development of perivascular neurogenic inflammation [21]. Melatonin, dose-dependently inhibits the LPS (*E. coli* isotype 0111: B4) induced increase in macrophage COX-2 protein expression. Melatonin has no influence on the expression pattern of the constitutive COX-1 isoform, thus revealing a specific effect on the inducible form. Melatonin in a concentration-dependent manner prevents the growth of PGE2, one of the major products of the COX pathway, induced by LPS incubation in macrophage cells [22]. Melatonin also has the ability to inhibit the enzyme nitric oxide synthase, modulates the secretion of serotonin and dopamine, which are substances related to the pathophysiology of migraine [21]. Melatonin also prevents LPS-induced iNOS growth in macrophages. However, the effectiveness of melatonin in inhibiting this enzyme is small. Melatonin at pharmacological concentrations prevents the increase of NO during LPS incubation, confirming the inhibitory effect on iNOS [22]. CGRP is a marker of trigeminal inflammation, implicated in the vasodilatation that occurs during a migraine attack [21]. In addition, CGRP inhibits gastric acid secretion and may inhibit food intake [23]. Melatonin can inhibit the release of CGRP and thus regulate blood flow in the brain [21]. Melatonin intensifies the inhibitory effect of GABA by potentiating the GABA-A receptor mediated response. It is likely that the GABAergic system mediates the hypnotic effect of melatonin. Reduction in melatonin concentration can lower the pain activation threshold, usually inhibited by GABAergic transmission. In addition, melatonin may affect the concentration of intracellular calcium, thus lowering its concentration affects the tension and reactivity of the brain vessels [24,25]. Melatonin inhibits stimulated dopamine release in the rat hypothalamus and rabbit retina. This inhibition is associated with suppression of calcium uptake by the stimulated tissue. Bidirectional effects of melatonin on calcium uptake in the hypothalamus have been documented at different times of the day [15]. Melatonin receptors have been located in the arteries of the brain, and melatonin itself may also modulate the function of serotonin 5HT2 receptors in the vessels [24,25]. However, the mechanism of the analgesic effect of melatonin has not yet been explained. One of the studies conducted suggested that melatonin may exert an analgesic effect by inducing (as an agonist) δ opioid receptors. Another study, on the other hand, did not show a direct relationship between melatonin and opioid receptors, while the influence of melatonin on the increase of β-endorphin release was postulated [25,26,27].

One of the main centers in the central nervous system that receives sensory stimuli from the gastrointestinal tract is the nucleus tractus solitarius (NTS) in the brain stem, which is located close to the trigeminal nucleus caudalis (TNC)—one of the major centers involved in the pathophysiology of migraine. The NTS receives impulses from the sympathetic and parasympathetic fibers of the gastrointestinal tract. Central sensitization, mediated by repetitive unpleasant gastrointestinal stimuli, may result in an expansion of the receptive field, lower excitability thresholds, and the potentiation of responses in the NTS. Consequently, through NTS-TNC interconnections, the TNC can also receive frequent pulses, thereby spreading neurogenic inflammation in the dura mater. These anatomical and pathophysiological relationships may explain the comorbidity of gastrointestinal disorders and migraine. In patients with gastrointestinal disorders, the brainstem is more sensitive and the trigemovascular system more prone to activation, which is probably why they suffer more from headaches [20].

It was noted that prodromal symptoms of migraine are related to hypothalamic dysfunction related to structures that have connections with the pineal gland and the ability to modulate melatonin secretion depending on changing environmental conditions. The hypothalamus also has connection pathways with the nuclei of the trigeminal nerve. Disturbances of the retina-hypothalamus-pineal axis play an important role in the pathophysiology of migraine. Seasonal and circadian oscillations in biological rhythms are primarily generated by the suprachiasmatic nuclei of the hypothalamus, and melatonin is the key hormone in this pathway [21,28].

The results of some clinical studies have addressed the link between the nocturnal melatonin secretion level and migraine [14].

**Figure 1:**
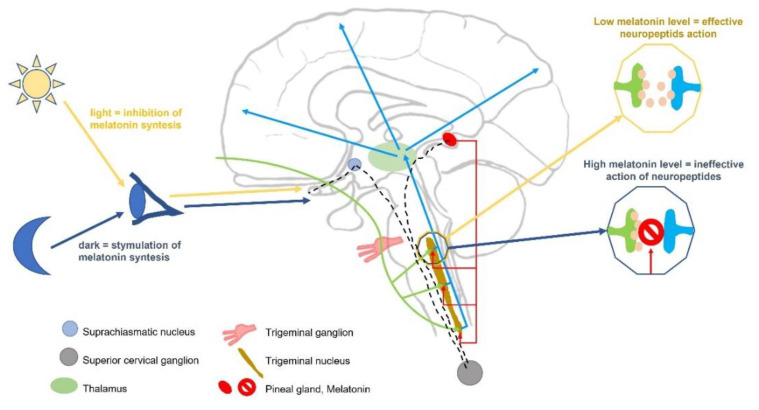
The potential role of melatonin in the pathogenesis of migraine modified according to: [29,30].

The potential role of melatonin in the pathogenesis of migraine is schematically shown in Figure 1.

Dietary factors may play a role in the pathophysiology of migraine. Diet can influence the modulation of neuroreceptors, neuropeptides and some ion channels, and also influence the sympathetic nervous system and the brain’s glucose metabolism by inducing inflammation, release of nitric oxide and vasodilation [31]. Certain foods such as chocolate, caffeine, milk, nuts, citrus fruits, monosodium glutamate, nitrites, aspartame, fatty foods, cheese, and alcoholic beverages have been identified as commonly provoking migraine attacks. Moreover, it has been observed that patients with triggers for migraine attacks are more prone to functional disability related to migraine [32]. The management of migraine patients should involve strategies to avoid triggers of migraine based on the needs of the individual patient. Diets high in fat, carbohydrates, or caffeine activate the sympathetic nervous system, which can trigger migraines. Dietary interventions such as diets high in folate, low-fat, high-omega-3, low-omega-6 diets, ketogenic diets, and low sodium diets reduce the frequency of migraine attacks [33]. The theory that diet-induced weight loss prevents headaches is also under consideration because obesity is associated with both episodic and chronic migraine, especially in younger people aged 20–55 [31]. The terminology “gut-brain axis” indicates a two-way relationship between the digestive system and the central nervous system. Migraine has been shown to be associated with certain gastrointestinal disorders, such as Helicobacter pylori infection, irritable bowel syndrome and celiac disease [23]. Evidence regarding the evolving role of the gastrointestinal microbiota in the gut-brain axis suggests that unbalanced gut flora (i.e., dysbiosis) is associated with migraine [34]. An inflammatory immune response with dysbiosis and increased intestinal permeability has been described in patients with migraine, and changes in the intestinal microflora may be a strong mediator in migraine [35,36].

## 3. Determinations of Melatonin Level in Episodic Migraine

Migraine, in its various forms, affects as much as 12–20% of the world’s population, contributing to a significant deterioration of the quality of life. Most often it occurs as episodic migraine [37]. According to the International Classification of Headache Disorders third edition diagnostic criteria from 2018 [38], migraine is a recurrent headache disorder manifesting in attacks lasting 4–72 h. Typical characteristics of the headache are unilateral location, pulsating quality, moderate or severe intensity, aggravation by routine physical activity and coexistence of nausea or/and photo/phonophobia. By definition, episodic migraine is characterized by headaches that occur on fewer than 15 days per month [39].

One of the first studies to assess melatonin levels in migraine patients, conducted in 1989 by Claustrat and colleagues, showed lower levels of melatonin in the serum collected at 23:00 in migraine patients compared to the control group. At the same time, it was observed that the concentration of melatonin itself was significantly lower in patients during the migraine attacks, compared to the interictal period [40]. However, the results of the Masruha et al., study from 2008 [41], with a group of 146 migraine patients, are different. The concentration of the melatonin metabolite (6-sulfatoxymelatonin) was determined using the ELISA method in the samples of the night urine (collection between 8:00 a.m. and 8:00 p.m.). No differences were observed between the melatonin concentration in the migraine group in the interictal period and the control group (people without headaches). Significantly lower melatonin concentration was noted in the group of patients who experienced a migraine attack during the tests, compared to the patients in the interictal period and in the control group. Moreover, no significant differences in melatonin concentration were observed when comparing the group of migraine patients with and without aura. A study by Zduńska et al. [42] also showed no statistically significant differences between the values of melatonin concentrations in patients with episodic migraine in the interictal period and patients without headache. Moreover, the courses of melatonin profiles in the group of migraine patients and in the control group showed no statistically significant interactions. It was observed that the median melatonin values were lower in the migraine group, but the differences between the groups were not statistically significant. On the other hand, in 2017, Kozak et al. [43] published a study in which melatonin levels and sleep quality were assessed in patients with migraine. Melatonin was measured at 1.00 a.m. The time between the last migraine attack and the sampling of blood was at least 48 h. Melatonin levels were lower in patients with migraine compared to the group without headaches, and this relationship was statistically significant. A study by Brun et al. [44] was carried out in a group of women with menstrual migraine. The concentration of melatonin in samples of nocturnal urine was measured during the entire menstrual cycle in migraine patients and a control group. This concentration was significantly lower in the group of migraine women. It was also observed that in the group of migraine patients—in contrast to the control group—there was no physiological increase in melatonin concentration in the luteal phase of the cycle. Similar relationships were also found in the study by Murialdo [45]—the concentration of melatonin in urine in samples taken during headache episodes was lower compared to the measurements in the painless period.

## 4. Determinations of Melatonin Level in Chronic Migraine

Chronic migraine develops in individuals with episodic migraine at a rate of about 2.5% per year. According to the International Classification of Headache Disorders third edition diagnostic criteria [38], chronic migraine is defined as headache occurring on 15 or more days/month for more than 3 months, which, on at least 8 days/month, has the features of migraine headache. However, diagnostic criteria distinguishing episodic from chronic migraine continue to evolve. Chronic migraine is associated with a substantially greater personal and societal burden, more frequent comorbidities, and possibly with persistent and progressive brain abnormalities [39].

In a study by Peres et al. [46] the circadian rhythm of melatonin secretion in patients with chronic migraine was assessed by measuring the concentration of melatonin in the serum between 7:00 p.m. and 7:00 a.m. A delay in the nocturnal peak of melatonin secretion was observed in the group of patients with chronic migraine, but there was no statistically significant difference between the concentration of melatonin in the study and control groups. Significantly lower melatonin levels were observed in patients with chronic migraine and coexisting insomnia. Bruera et al. [47] measured melatonin levels in patients with various types of idiopathic headaches, including REM sleep, NREM, and wakefulness. Abnormal levels of melatonin (no characteristic increase in REM sleep and significantly lower levels after waking up compared to the control group) were noted only in the group of patients with chronic migraine and chronic tension type headache.

Table 1 summarizes the studies, the results of which are discussed in the article.

## 5. Melatonin in the Treatment of Migraine

Melatonin essentially improves sleep quality, and consuming melatonin-rich foods helps you fall asleep. Foods high in melatonin include eggs and fish from animal products, and nuts, certain types of mushrooms, grains, legumes, and seeds from plant products. Increasing the intake of melatonin in the diet seems to be a good solution for the elderly, as its concentration decreases with age. It has been shown that consuming melatonin-rich foods can have health effects by increasing circulating melatonin, and serum melatonin levels can be increased by consuming foods that contain melatonin. Melatonin has also been used successfully as a drug or dietary supplement to regulate sleep disorders or jet lag syndrome [48].

The discovery of the role of melatonin in the pathophysiology of migraine is very important due to its possible use in chronic therapy or acute therapy. Current melatonin preparations typically include immediate-release melatonin, sustained-release melatonin, and melatonin receptor agonists (Agomelatine, Ramelteon, and Tasimelteon). Various forms and doses of melatonin-containing drugs have been examined in studies [9].

Ebrahimi-Monfared [49] investigated the therapeutic effect of melatonin compared to valproic acid in the prophylactic treatment of chronic migraine. All patients received basic treatment with nortriptyline (10–25 mg) and propranolol (20–40 mg). Additionally, patients were given (randomized double-blind, placebo-controlled trial) valproic acid at a dose of 200 mg or melatonin 3 mg or a placebo. Supplementary treatment with melatonin was shown to be more effective than placebo, and had similar clinical efficacy to valproic acid but better tolerability. In the future, melatonin may prove to be an effective substitute for valproic acid in the prevention of chronic migraine. On the other hand, Ali et al. [50] conducted a prospective controlled study and investigated the effectiveness of 5 mg melatonin versus 40 mg propranolol twice daily for 3 months in migraine patients. Treatment was effective, but no difference was found between the two groups. Gonçalves [51] compared the use of 3 mg of melatonin with 25 mg of amitriptyline and a placebo. Melatonin was found to be more effective than placebo and similarly effective to amitriptyline in reducing the frequency of headaches in the prophylactic treatment of migraine. At the same time, melatonin was better tolerated by patients than amitriptyline. The aim of the study by Bouge [52] was to evaluate the potential efficacy of melatonin in preventing primary headache. Patients diagnosed with chronic migraine and chronic tension type headache were prescribed oral melatonin at a dose of 4 mg, 30 min before bedtime, for six months. There was a statistically significant reduction in the incidence of headache between baseline and final follow-up after six months of treatment. Alstadhaug et al. [53] conducted the only study in which a 2 mg dose of melatonin was used in the prophylactic treatment of migraine in patients with a seizure frequency of 2–7 per month over a period of 2 months. The study showed that taking this dose of sustained-release melatonin did not reduce the frequency of headache attacks compared to placebo. In the remaining studies, 3 mg of melatonin was used. In an open-label clinical study conducted by Peres [54], melatonin at a dose of 3 mg was shown to be effective in reducing the intensity of headache. In a study conducted in a small group of women, an intravenous infusion of melatonin was used during a migraine state. The nocturnal profile of melatonin was assessed and the kinetics of therapeutically administered melatonin was investigated. The melatonin profile was disturbed in 3 migraine patients. Most of the patients had relieved or reduced headache [13].

The aim of the network meta-analysis (NMA) carried out by Ping-Tao Tseng et al. [37] was to compare the efficacy of exogenous melatonin supplementation in patients with episodic migraine. Based on the analysis, it was shown that only oral melatonin 3 mg/d (immediate-release) at bedtime was associated with significantly better improvements in frequency of migraine days than placebo, while oral melatonin at a dose of 3 mg/d (immediate release) at bedtime turned out to be the most beneficial in therapy. Pacheco [55] reviewed studies using melatonin in the prophylactic treatment of migraine. He found only a few clinical trials, with poor methodological quality concerning melatonin for primary headaches. It was concluded that the available evidence was not sufficient to support the use of melatonin in clinical practice for examined populations.

Current research into the use of melatonin agonists in the treatment of migraine is promising. Agomelatine is an agonist of the MT1 and MT2 melatonin receptors and a selective antagonist of the 5-HTC-2C receptor. Guglielmo et al. [56] reported two cases of patients with episodic migraine (one patient with comorbid depression, the other without comorbidities) successfully treated with agomelatine 50 mg/day. A reduction in the frequency of migraine attacks was observed in both patients. Currently, Tasimelteon and Ramelteon [57] are not extensively researched. Both Ramelteon and Tasimelteon are non-selective melatonin receptor agonists. Only a case report of a patient with chronic migraine and coexisting insomnia who was started on ramelteon at a dose of 8 mg/day has been published. After 2 weeks of observation, sleep time increased to 4–5 h with less fragmentation, and the severity of migraine assessed on the MIDAS scale began to decrease. After 6 months of treatment, the severity of migraine continued to subside with stable sleep quality. Tasimelteon, as the only agonist that has a higher relationship for the MT2 receptor than for the MT1, may show promise in the prophylaxis of migraine. Published data underline the importance of the MT2 receptor in insomnia, anxiety, depression, pain, and central nervous system (CNS) neurodegenerative disease as the MT2 receptor is predominantly found in the CNS [58].

## 6. Summary and Conclusions

The results of studies available in the literature assessing melatonin levels in migraine patients are variable and depend on many factors. Probably, the selection and size of the study group are important. The results also depend on the nature of the headache/migraine (chronic or episodic) and disease period during which melatonin concentration was measured (during a headache attack or in the interictal period). In several of the cited studies, no statistically significant differences were found between melatonin levels in migraine patients during the pain-free period compared to the group without headaches. The question is how to explain the occurrence of decreased melatonin concentration only in patients who experienced a migraine attack during the tests? Perhaps hypothalamic dysfunction occurs only during the migraine attack itself and in the prodromal phase, therefore melatonin levels do not decrease in the interictal period. These findings suggest the role of the hypothalamus in the pathophysiology of migraine. It should be underlined that in several studies, the decreased concentration of melatonin occurred only in patients with chronic migraine. Perhaps only a chronic migraine headache or a high frequency of attacks disturbs the function of the hypothalamus and haves an inhibitory effect on melatonin secretion [28,47,59]. In the group of patients with migraine, it is likely that the disease process disturbed the release of melatonin. In explaining the causes of this phenomenon, several mechanisms have been taken into account including local abnormalities in the functioning of the sympathetic system innervating the pineal gland, hypersensitivity of the retinal-hypothalamic pathway, and impaired functioning of the hypothalamic suprachiasmatic nucleus in patients with migraine [60].

There is no conclusive evidence to support the effectiveness of melatonin in treating migraine, although according to data from controlled and uncontrolled studies and clinical observations, there appears to be a strong association between melatonin and migraine headache relief. The variability in results may depend on the dose of melatonin used (3 mg vs. 2 mg) and the form of the drug (immediate release vs. prolonged release), duration of therapy (3 months and longer vs. 2 months), the number of patients and measures of outcomes (headache days and frequency of attacks vs. frequency of attacks) [9].

Studies have confirmed the effectiveness of 3 mg immediate release melatonin and 4 mg prolonged release melatonin, and the melatonin receptor agonist (Agomelatine) 25 mg. More research is needed to determine the optimal form and dose of melatonin-containing medications for migraine prevention. Moreover, melatonin was ineffective in a 2-month study, therefore 3 months or more may be an appropriate duration of migraine treatment. The excellent tolerance of melatonin makes it a potentially favorable candidate for migraine therapy. Adverse events are generally few and mild [9,61]. Large-scale, randomized, multicentre studies are necessary to assess whether melatonin is actually beneficial and well tolerated, and to determine the optimal dosage and formulate the principles of melatonin use in the treatment of migraine [21].

Of course, melatonin has not yet found its place in migraine and other headache management recommendations, but it can be complementary to standard and recommended treatment. Certainly, its role and effectiveness in migraine should be assessed in reliable, controlled clinical trials. However, it seems that side effects of melatonin (if any) do not limit its use.

## Figures and Tables

**Table 1:** Studies investigating the relationship between melatonin levels and migraine.

Reference	Method of Study	Patients No	Diagnosis	Melatonin Test Method	Outcome
Claustrat et al., 1989 [40]	case control	93 + 46	migraine + control	serum melatonin RIA	Lowering the plasma melatonin level in the entire migraine population compared to the control group
Masruha et al., 2008 [41]	case control	146 + 74	migraine + control	urine aMT6s ELISA	Urinary aMT6s concentration in patients with migraine attack during sample collection was significantly lower than in migraine patients without headache and in the control group. There was no significant difference in the urinary aMT6s concentration in migraine patients without pain on the day of their urine samples collection and in controls
Zduńska et al., 2021 [42]	case control	29 + 29	migraine + control	serum melatonin RIA	No statistically significant differences between melatonin concentrations in patients with episodic migraine in the interictal period and control group
Kozak et al., 2016 [43]	case control	55 + 57	migraine + control	serum melatonin ELISA	The level of melatonin was significantly lower in the migraine patients than in control
Brun et al., 1995 [44]	case control	10 + 9	menstrual related migraine + control	urine melatonin RIA	The mean nocturnal melatonin excretion throughout the cycle was significantly lower in women with migraine than in the control group. In the control group, the excretion of melatonin increased significantly from the follicular to luteal phase, while in migraine patients, this phenomenon was not observed
Murialdo et al., 1994 [45]	case control	12 + 8	menstrual related migraine + control	urine melatonin RIA	Nocturnal urinary melatonin excretion was significantly lower in migraine patients than in the control group. The increase in urinary melatonin excretion during the luteal phase was less marked in migraine patients. The decrease in melatonin excretion increased even more during the headache
Peres et al., 2001 [46]	case control	17 + 9	chronic migraine + control	serum melatonin RIA	Delayed nocturnal peak of melatonin level in chronic migraine patients as well lower melatonin levels in chronic migraine patients with insomnia
Bruera et al., 2008 [47]	case control	30 + 10	various idiopathic headaches + control; during NREM and REM sleep as well waking up	serum melatonin RIA	Significantly lower levels of melatonin after waking up and no characteristic increase of REM sleep, compared to the control group, were found only in patients with chronic migraine and chronic tension headache

## Data Availability

Not applicable.
